# Nutritional Capability of and Substrate Suitability for *Pseudogymnoascus destructans*, the Causal Agent of Bat White-Nose Syndrome

**DOI:** 10.1371/journal.pone.0078300

**Published:** 2013-10-21

**Authors:** Daniel B. Raudabaugh, Andrew N. Miller

**Affiliations:** 1 Department of Plant Biology, University of Illinois, Urbana, Illinois, United States of America; 2 Illinois Natural History Survey, University of Illinois, Champaign, Illinois, United States of America; California Department of Public Health, United States of America

## Abstract

*Pseudogymnoascus destructans*, the causal agent of bat white-nose syndrome, has caused nearly six million deaths in North American bats since its introduction into the United States in 2006. Current research has shown that caves can harbor *P. destructans* even after the infected bats are removed and bats no longer visit or inhabit previously infected caves. Our research focuses on elucidating reservoir requirements by investigating the nutritional capabilities of and substrate suitability requirements for six different *P. destructans* isolates from various localities including Illinois, Indiana, New York (Type specimen), and Pennsylvania. Enzyme assays implicate that both urease and b-glucosidase appear to be constitutive, lipase and esterase activity were more rapid than proteinase activity on 6% gelatin, gelatin degradation was accompanied by medium alkalinization, the reduction of thiosulfate generated hydrogen sulfide gas, chitinase and manganese dependent peroxidase activity were not visually demonstrated within eight weeks, and keratinase activity was not evident at pH 8 within eight weeks. We demonstrate that all *P. destructans* isolates are capable of growth and sporulation on dead fish, insect, and mushroom tissues. Sole nitrogen source assays demonstrated that all *P. destructans* isolates exhibit Class 2 nitrogen utilization and that growth-dependent interactions occur among different pH and nitrogen sources. Substrate suitability assays demonstrated that all isolates could grow and sporulate on media ranging from pH 5–11 and tolerated media supplemented with 2000 mg/L of calcium and 700 mg/L of three separated sulfur compounds: thiosulfate L-cysteine, and sulfite. All isolates were intolerant to PEG-induced matric potential with delayed germination and growth at −2.5 MPa with no visible germination at −5 MPa. Interestingly, decreasing the surface tension with Tween 80 permitted germination and growth of *P. destructans* in −5 MPa PEG medium within 14 days suggesting a link between substrate suitability and aqueous surface tension altering substances.

## Introduction

The psychrophilic fungus *Pseudogymnoascus* (*Geomyces*) *destructans* (Blehert & Gargas) Minnis & D.L. Lindner (Pds) has been identified as the causal agent of bat White Nose Syndrome [1], [2] which has been implicated in almost six million North American bat deaths since its initial outbreak in 2006 [3]–[5]. Current scientific data suggests that Pds originated from Europe and since its introduction to New York State in 2006, has spread to 22 states and 5 Canadian provinces [2]. *Pseudogymnoascus destructans* is known to cause severe skin lesions and cutaneous necrosis in bats during hibernation, which has been suggested to disrupt wing circulation, cutaneous respiration, and promote dehydration [6], [7]. *Pseudogymnoascus destructans* can infect hibernating bats because the host's internal body temperature is reduced to slightly above ambient temperatures (ca. 2 to 10°C) and the bat immune system is reported to be suppressed [3], [4]. If an infected bat survives hibernation, the bat's body temperature and immune system return to normal and Pds is eradicated from the host's epidermal layers [8]. Recent research has shown that Pds is capable of surviving in caves no longer populated by infected hibernating bats [9], which points to the potential for infected caves to act as Pds reservoirs. Many aspects of Pds (geographic distribution, temperature dependent growth, and genomics) have been thoroughly researched, but no comprehensive research thus far has been conducted on the basic biology of Pds.

Limited studies have shown Pds to be temperature sensitive, produce numerous enzymes under laboratory conditions, and contain several dual virulence factors. Temperature effects on the growth and morphology of Pds demonstrated that Pds has active growth from 0 to about 20°C, with optimal growth from 12.5 to 15.8°C, and an increased occurrence of hyphal stress-related structures (hyphal thickening, presence of arthrospores and chlamydospore-like structures) above ca. 15°C [10]. *In vitro*, Pds has been shown to produce β-glucosidase, N-acetyl-β-glucosaminidase, acid and alkaline phosphatases, esterase/esterase lipase/lipase, leucine and vailine arylamidase, naphthol-AS-B1-phophohydrolase, various proteinases (albumin/casein/gelatin), and urease, while no enzymatic activity was indicated for cystine arylamidase, α-chymotrypsin, alpha/beta galactosidase, trypsin, β-glucoronidase, α-fucosidase, and α-mannosidase [11]. Notably, some of these enzymes present in Pds (urease, proteinase (aspartyl), and superoxide dismutase) are found in other pathogenic fungi [12], [13] and are considered dual virulence factors [14].

Our research goals are to obtain a more comprehensive understanding of the reservoir requirements for Pds by focusing on three key questions: 1) What is the nutritional capability of Pds? 2) Which cave substrates are suitable for Pds growth? and, 3) Does the increase in water surface tension at cold temperatures effect substrate suitability? To answer our first two questions we categorized carbon containing cave resources into four groups of substrates: keratinaceous, chitinaceous, cellulosic, and protein/lipid rich substrates. In addition to common carbon containing cave resources, we investigated other important environmental factors including nitrogen utilization, pH tolerance, and tolerance to various environmental levels of calcium and sulfur compounds. To elucidate how the increase in water surface tension at cold temperatures effect substrate suitability we examined the growth of Pds under known levels of matric-induced water stress and compared those results to growth under identical conditions containing a surface tension reduction agent. Lastly, we preformed an additional assay to investigate if Pds could produce its own biological surfactant.

## Methods

Automated systems for characterizing fungal metabolic functions are becoming more common in research. One such system, the Biolog microplate is widely known for its bacterial plates but also offers a fungal carbon source specific plate (FF GEN II plate). Each test well in the Biolog FF GEN II plate contains one carbon source where color development and turbidity are measured. For our carbon assays, we decided to use a more traditional approach in order to monitor secondary reactions, which are equally important for understanding how substrates are degraded. We determined that traditional culture techniques would also allow for greater flexibility by quickly adding, combining, or altering environmental resources, which can influence metabolic function.

### Culture and assay conditions

Six Pds cultures were examined; three from Illinois (ILLS69284, ILLS69285, ILLS69286), and one each from Indiana (GdINMSC7), New York (MYA-4855, Type culture) and Pennsylvania (ILLS69283). All cultures were obtained from publicly available mycological culture collections at the University of Illinois, Western Illinois University, ATCC, and Lock Haven University. All isolates were maintained in 24 hour darkness on Difco Sabouraud's Dextrose Agar (SDA) at 7°C. To promote conidiation, 200 mg/L MnSO_4_ (Sigma Aldrich (SA)) was added to the stock culture medium. Unless noted, all media were sterilized at 121°C under 15 PSI for 15 minutes, wrapped with Parafilm (Fisher Scientific (FS)) after inoculation, and all plates were inverted two days after inoculation. All assays were inoculated with actively growing mycelium and replicated twice at different times using two replicates each time. All assays were incubated in 24 hour darkness; carbon, sulfur, pH, calcium, and nitrogen assays were conducted at 9°C, while surface tension, matric potential, and the biosurfactant assays were conducted at 10°C. All Petri plate assays contained ca. 20 ml of media unless otherwise noted. Glassware was cleaned by immersion in 30% glacial acetic acid or 6 M HCl for at least 6 hours, rinsed with distilled water, and autoclaved twice prior to use.

### Analyses

Matric potential was adjusted using polyethylene glycol (PEG) 8000 (FS) following the equation; ψ = 1.29[PEG]^2^T-140[PEG]^2^-4.0[PEG] where [PEG] = gram of PEG 8000 per gram of water and T = temperature (°C) [15]. Surface tension was measured with a stalagmometer using fungal free (centrifuged at 14000 rpm for 2 min) 10°C media following the equation 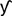
_L_ = (m_L_/m_Water_)
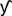
_water_ where m_L_ and m_water_ are the average weight of 50 drops and 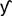
_water_ = 74.23×10^−3^ N/m. Spore count was conducted using an improved Neubauer hemocytometer, pH was measured with a Milwaukee MW102 pH/temperature meter, microscopic evaluations were conducted using an Olympus SZX12 stereo microscope, cave soil matric potential was determined following two equations; Wf<45.3%: Log(h) = 5.327-0.0779Wf or Wf>45.3%: Log(h) = 2.412-0.0135Wf where Wf is filter paper water content (%) and h = matric potential (KPa) [16]. One-way ANOVA with Tukey's post test was performed using GraphPad Prism version 5.03 for Windows to determine statistical significance for the tolerance and biosurfactant assays.

### Whole carbon source assays

Freeze-dried *Locusta migratoria* L., fresh *Poecilia* sp., and dried *Lentinula edodes* (Berk.) Pegler were placed into glass Petri plates, autoclaved, and inoculated with an autoclaved cotton swab that was pre-moistened in a 1% NaCl solution and rubbed over ca. 5 mm×5 mm sporulating Pds culture. Fresh exoskeletons of *Pleoticus muelleri* Bate (50 g) were subjected to a demineralization step [17], which consisted of two, 300 ml ice cold 0.25 M HCl treatments (one for 5 minutes and the second for 35 minutes) followed by neutralization with distilled water. Additional fresh exoskeletons were subjected to the above demineralization step and a deproteination step [17], which incorporated three separate 100 ml (1.0 M NaOH) treatments at 95°C; the first and second treatments were 2 hours, and the third was for 1 hour followed by neutralization with distilled water. The demineralized and deproteinated exoskeletons were placed in glass Petri plates, autoclaved, and inoculated as above. All plates were visually monitored twice a week for the presence of mycelial growth and conidiation.

### Carbon source enzymatic assays

#### Proteinase activity

Gelatin degradation and alkalinization assays medium consisted of distilled water containing 3% or 6% gelatin (Knox original unflavored) and 0.002% phenol red (SA) as the pH indicator [18]. The 3% gelatin medium was adjusted to pH 6.0±0.1 and the 6% gelatin medium was adjusted to pH 7.0±0.1 with 3% KOH prior to sterilization. Each sterilized gelatin medium (150 µl) was pipetted into a separate UV-sterilized 96-well standard microplate and each test well was inoculated with a BB size pellet of scraped mycelium. The 96-well plates were covered with microtiter plate sealing film, incubated, and monitored daily for gelatin degradation (liquification) and pH change (color change from yellow below pH 6.8 to red/pink above pH 8.2). Our gelatin medium differed from [18] since the only carbon and nitrogen source was gelatin. We did not include other carbon or nitrogen sources because dermatophytes are reported to use proteins for their sole source of carbon and nitrogen [19]. We used 3% gelatin for the detection of low gelatinase activity and alkalinization, and 6% gelatin medium for gelatinase activity and to ascertain if there was medium acidification prior to medium alkalinization.

#### Keratinase activity

Alpha-keratin basal medium (ca. pH 8) consisted of 0.1% glucose (SA), 0.2% yeast extract (Difco), 0.1% KH_2_PO_4_ (FS), 0.02% MgSO_4_•7H_2_O (FS), 1% Na_2_CO_3_ (SA, autoclaved separately and added to achieve pH 8), and 1.5% agar (SA) L^-1^ distilled water [20]. Keratin azure (SA) was cut into small ca. 3 mm length sections, autoclaved separately in a dry beaker and a portion of the cooled (55°C) basal medium was added to the keratin azure to produce a 1% keratin azure overlay medium. Using sterile techniques, 5 ml of basal medium was added to 10 ml autoclaved glass test tubes. After the basal medium solidified, 1 ml of the 1% azure keratin overlay medium was pipetted into the glass test tubes. Each test tube was inoculated with a BB size pellet of scraped mycelium. Test tubes were sealed with Parafilm and visually monitored for dye release into the basal medium at week 6 and week 8. We did not include carboxymethyl cellulose in our medium as a means to eliminate an extraneous carbon source, which would not be found on natural keratin substrates. Keratin degradation can be influenced by environmental reducing agents [21], so two additional iterations of the alpha-keratinase experiment were conducted using the keratin basal medium, but supplementing one iteration with 0.5 mg/L Na_2_S_2_O_3_·5H_2_O and supplementing the other with 0.5 mg/L Na_2_SO_3_. Both iterations were conducted at pH 7.5.

Beta-keratin derived assay medium consisted of soluble keratin; 10 g of white chicken feathers where refluxed at 100°C in 500 ml dimethyl sulphoxide for 2 hours followed by precipitation using 1000 ml of -80°C acetone [22]. The precipitated keratin was filtered using sterile Whatman #1 filter paper and washed four times (50 ml each) with sterile distilled water. The precipitate was then added to filter sterilized 0.1 M phosphate buffer at pH 7 to obtain a 0.06% soluble keratin solution. To this solution, 0.5 g MgSO_4_•7H_2_O, 0.5 g FeSO_4_ (SA) and 5 mg ZnSO_4_ (SA) L^-1^ was added, autoclaved, and portions of the resulting wet keratin mat were placed in sterile glass Petri dishes. The keratin mats were inoculated in the same manner as the whole carbon source assays. All plates were monitored twice a week for mycelial growth and conidiation. This method differs from [22] in that we did not precipitate our keratin in −90°C acetone because this extreme precipitation temperature would only affect the resulting keratin yield, and we buffered our keratin to pH 7 to avoid keratin degradation during autoclaving [21]. Our growth medium is similar to the liquid culture technique found in [22], except we used a keratin mat instead of keratin particles suspended in liquid, which avoids potential aeration issues associated with some liquid culture techniques [23].

#### Chitinase activity

Colloidal chitin was obtained as follows: 20 g of crab shells were ground into a powder, acid digestion at room temperature with 150 µl of 12 M HCl for 1 hour under constant stirring, precipitation in ice cold water, vacuum filtration (coffee filter: two layers), neutralization by wrapping the precipitate in multiple layers of coffee filters and floating the wrapped precipitate in two changes of 500 ml of distilled water at room temperature for a total of 24 hours, unwrapped, autoclaved, and stored in a sealed container at 10°C until needed [24]. Colloidal chitin media consisted of 2% moist colloidal chitin (prepared from crab shells as above), 0.7 g K_2_HPO_4_ (SA), 0.3 g KH_2_PO_4_, 0.5 g MgSO_4_•7H_2_O, 0.5 g NaCl (FS), 0.5 g FeSO_4_, 0.2 mg ZnSO_4_, 0.1 mg MnSO_4_ (SA), 5 µg thiamine chloride (SA), and 100 µg Biotin (SA), which was filter sterilized (0.20 µm) and added to 55°C media and 2% agar L^-1^ distilled water. A basal version of this medium (containing no chitin) was poured into the Petri plate and allowed to solidify after which 2 ml of colloidal chitin medium was pipetted on top, inoculated with a 5 mm agar plug, and examined twice per week for the presence of a clearing zone around the colony. Our extraction differed from [24] only in the neutralizing step; we wrapped the chitin precipitate in coffee filters and floated the precipitate in water to maximize the resulting yield and prolong the neutralization step. To achieve clear results, our assay utilized a chitin free-basal medium in conjunction with the 2% chitin medium as an overlay.

#### Cellulase and ligninase activity

Beta-glucosidase activity was determined using esculin plus iron agar: 5 g L-tartaric acid diammonium salt (SA), 0.5 g MgSO_4_•7H_2_O, 1 g K_2_HPO_4_, 0.1 g yeast extract, 0.001 g CaCl_2_ (SA), 2.5 g Esculin sesquihydrate (Alfa Aesar), and 8 g agar L^-1^ distilled water [25]. The medium was adjusted to pH 7.0±0.1 with 3% KOH prior to sterilization. Aqueous ferric sulphate (2%) was added to 55°C medium at a ratio of 1 ml per 100 ml of media. The medium was then poured into Petri plates and inoculated with a 5 mm diameter agar plug and evaluated twice per week for the appearance of a brownish black precipitate. Manganese-dependent peroxidase was assayed by supplementing 39 g Difco Potato Dextrose Agar (PDA) with 100 mg/L and 200 mg/L MnSO_4_ L^-1^ distilled water [26]. The medium was sterilize, inoculated as above, and examined twice per week for a black precipitate.

#### Lipase and esterase activity

Rhodamine B agar medium consisted of 8 g tryptone (Difco), 4 g NaCl, and 10 g agar L^-1^ distilled water [27]. The medium was adjusted to pH 7.0±0.1 with 3% KOH prior to sterilization. The medium was cooled to 55°C prior to the addition of 31.25 ml of sterile lipid (either lard or olive oil) and 0.001% wt./vol. rhodamine B (Acros). The medium was shaken vigorously for 1 minute to emulsify the lipid, poured into Petri plates, inoculated with a 5 mm diameter agar plug, and monitored twice a week for the appearance of orange fluorescence when exposed to UV light at 365 nm [28]. The Victoria Blue B and Tween 80 medium consisted of 10 g tryptone, 5 g NaCl, 0.1 g CaCl_2_ (SA), 0.01% wt./vol. Victoria blue B (Acros), 10 g Tween 80 (Acros, autoclaved separately), and 15 g agar L^-1^ distilled water [29]. The medium was adjusted to pH 7.0±0.1 with 3% KOH prior to sterilization. The medium was cooled to 55°C prior to the addition of Tween 80, shaken for 1 minute to emulsify the Tween 80, poured into Petri plates, inoculated with a 5 mm diameter agar plug, and examined twice per week for the presence of calcium soap (fatty acid) crystals. The original base media from [27], [28] used nutrient broth, which contains peptone (protein digest), beef extract and NaCl. We substituted nutrient broth with tryptone (protein digest) since it is a comparable nutritional source for Pds and the assay reaction was not compromised when compared to the positive control (*Serratia marcescens* Bizio).

### Nitrogen source assays

#### Urease activity

Stuart's urea broth base (0.1 g yeast extract, 9.1 g KH_2_PO_4_, 9.5 g Na_2_HPO_4_, 0.012 g phenol red L^-1^ distilled water (pH 6.8±0.2)) and modified Christensen's urea broth base (1.0 Tryptone, 2 g KH_2_PO_4_, 1 g dextrose (SA), 5 g NaCl, 0.012 g phenol red L^-1^ distilled water(pH 6.8±0.2)) without urea were used as control media. Filter sterilized (0.22 µm) urea (FS, 20 g L^-1^) was added to a portion of each cooled medium above to test for urease activity. An aliquot of each media (150 µl) with and without urea was pipetted into a separate UV-sterilized 96-well standard microplate well. For control purposes, only half of each of the medium types were inoculated with a BB size pellet of scraped mycelium. The 96-well plate was covered with microtiter plate sealing film and was monitored daily (visually) for a change in color (yellow to pink) in both the inoculated and control wells. Christensen's urea broth base was modified by the substitution of tryptone in place of peptone since it is a comparable nutritional source for Pds.

#### Nitrate, nitrite, ammonium, L-asparagine, and uric acid assays

The basal media consisted of 20 g D-glucose (SA), 2 g KH_2_PO_4_, 0.5 g MgSO_4_•7H_2_O, 0.5 g FeSO_4_, 0.2 mg ZnSO_4_, 0.1 mg MnSO_4_, 5 µg thiamine chloride, and 100 µg Biotin: 0.20 µm filter sterilized and added to 55°C media L^-1^ distilled water [30] and one of the following five nitrogen sources: 1) 3.07 g KNO_3_ (FS), 2) 2.0 g (NH_4_)_2_SO_4_ (FS), 3) 2.58 g KNO_2_ (SA), 4) 2.28 g L-asparagine monohydrate (FS), or 5) 1.275 g C_5_H_4_N_4_O_3_ (SA). After sterilization and addition of the micronutrient solution, each medium pH was adjusted using a saturated solution of NaOH and 12 M HCl. Assays were conducted at pH 5, 6, 7, and 8 for basal media containing nitrate, nitrite, L-asparagine, ammonium, or no added nitrogen source (control) and at pH 8 for uric acid. An aliquot of each medium (150 µl) was pipetted into a separate UV-sterilized 96-well standard microplate well. Each well was inoculated with a BB size pellet of scraped mycelium. The 96-well plate was covered with microtiter plate sealing film and monitored daily for mycelial growth.

### Substrate suitability assays

Since numerous fungal researchers use PDA as a base medium for chemical tolerance assays [31], [32], we utilized PDA for both calcium and sulfur tolerance assays. Tolerance was determined 25 days after media inoculation by measuring the radial diameter of each colony twice at 90° angles and averaging the two values. All replicate data were compiled and analyzed for statistical significance as previously described.

#### Calcium tolerance assay

Assay media consisted of 39 g Difco PDA medium supplemented with 500 mg/L, 1000 mg/L, and 2000 mg/L calcium chloride. Plates were inoculated with a 5 mm diameter agar plug, incubated, and assayed for tolerance as described above.

#### Environmental sulfur tolerance and hydrogen sulfide production assays

Assay media consisted of 39 g Difco PDA medium supplemented with 0.2 g FeSO_4_ and one of the following: Na_2_S_2_O_3_·5H_2_O (Baker), Na_2_SO_3_ (Mallinckrodt), or 97% L-cysteine (SA) at 100 mg, 300 mg, 500 mg, and 700 mg L^-1^ distilled water. After sterilization, Petri plates were inoculated with one 5 mm diameter agar plug, examined twice per week for a black precipitate, and assayed for tolerance as described above.

#### Medium pH assay

Our basal medium consisted of 9 g Malt broth (Difco), 2.25 g tryptone, and 18 g agar per 900 mL of distilled water [33]. One of the following buffer solutions (100 ml) was added after sterilization to achieve the following pH levels: pH 5 (6.9 g NaH_2_PO_4_•H_2_O (FS)), pH 7 (3.904 g NaH_2_PO_4_•H_2_O (FS) and 3.105 g Na_2_HPO_4_ (Macron)), pH 9 (0.318 g Na_2_CO_3_ and 3.948 g NaHCO_3_ (FS)), and pH 11 (5.3 g Na_2_CO_3_). Plates were inoculated with a 5 mm diameter agar plug and tolerance was evaluated as described above. The only modification of [33] was the substitution of tryptone in place of peptone since it is a comparable nutritional source for Pds.

#### Matric potential, surface tension, and surfactant production assays

The matric potential medium consisted of 0.2 g sucrose, 0.2 g D-glucose, 1 g KNO_3_, 1 g KH_2_PO_4_, 0.5 g MgSO_4_•7H_2_O, 0.5 g NaCl L^-1^ distilled water and adjusted to pH 7 with NaOH [34]. The medium was amended with PEG 8000 as described above to generate the following matric potentials: −0.07 MPa, −1 MPa, −2.5 MPa, and −5 MPa. After sterilization, 40 ml of each medium was inoculated with 500 µL of spores suspended in water (average spore count  = 3.1×10^7^±1.1×10^6^ per 500 µl), sealed with parafilm, and placed on shake culture at 100 rpm for 25 days. The assay was visually inspected weekly for germination and subsequent growth. Tolerance was qualitatively assayed as either visible growth or no visible growth at day 25. The current assay increased the speed of the shake culture from [34] to account for the high surface tension of water at low temperatures [35] and increased the medium pH to reduce mycelial aggregation [36]. The surface tension assay was identical to the matric potential assay except that the 500 µl spore suspension contained 1% Tween 80 (average spore count  = 4.1×10^6^±9.2×10^4^ per 500 µl) which was demonstrated in a similar study [36] to reduce water surface tension. The assay was visually inspected weekly for germination and subsequent growth. To determine if Pds produced extracellular biosurfactants, we repeated the matric potential experiment using two isolates (MYA-4855, GdINMSC7) in the −1 MPa conditions. After sterilization, 40 ml of the medium was inoculated with either 500 µL of spores suspended in water (average spore count  = 3.1×10^7^±1.1×10^6^ per 500 µl) or 500 uL of sterile water (control), sealed with parafilm, and placed on shake culture at 100 rpm. On day 15, fungal free filtrate was obtained and the surface tension was measured with a stalagmometer as previously described above.

## Results

### Carbon

**Figure 1:**
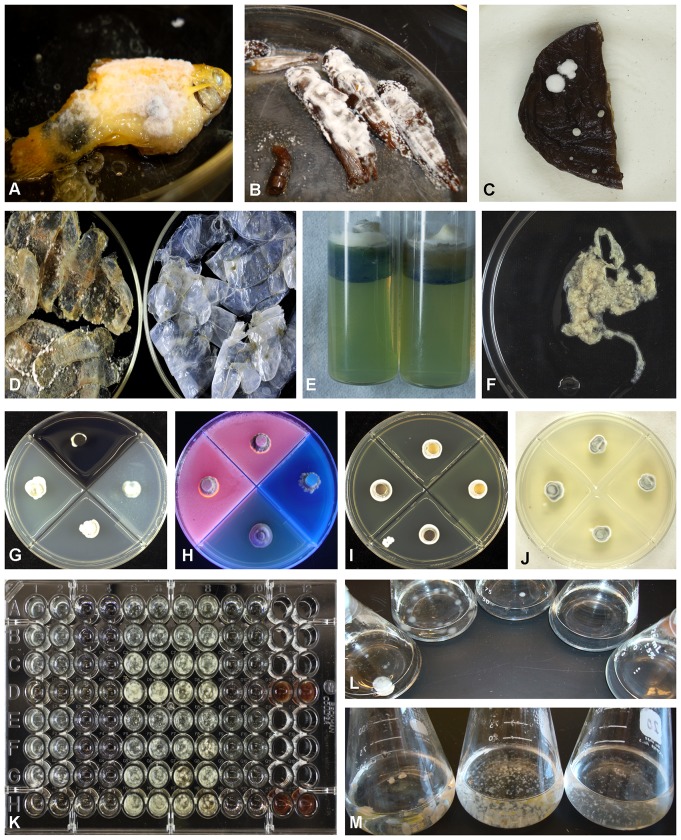
*Pseudogymnoascus destructans* growth and morphology. A) *growth on Poecilia* species. B) growth on *Locusta migratoria*. C) growth on *Lentinula edodes*. D) growth on demineralized shrimp exoskeletons (left) and demineralized/deproteinated exoskeletons (right). E) growth on keratin azure medium (no dye released). F) no growth on beta-keratin derived mat assay. G) growth on carbon media, clockwise from bottom: PDA, Mn-amended PDA, β-glucosidase (+ Rx), collidial chitin. H) growth on lipid and ester media, clockwise from bottom: SDA, Olive oil (+ Rx), Lard (+ Rx), Tween 80. I) growth on calcium media, clockwise from bottom: PDA, 500 mg/L calcium, 1000 mg/L calcium, 2000 mg/L calcium. J) growth on pH media, clockwise from bottom: pH 5, pH 7, pH 9, pH 11. K) growth on nitrogen media, left to right: columns 1–2 =  nitrate, columns 3–4 =  nitrite, columns 5–6 =  ammonium, columns 7–8 =  L-asparagine, columns 9–10 =  control with no nitrogen source, columns 11–12 =  uric acid. Rows A–D (MYA-4855), Rows E-H (ILLS69283), rows A, E (pH 5), rows B, F (pH 6), rows C, G (pH 7), rows D, H (ph 8). L) matric potential assay, left to right: growth at −0.07 MPa, −1 Mpa, and −2.5 Mpa; no growth at −5 MPa and −7.5 Mpa. M) surface tension reduction assay with the addition of 0.0125% Tween 80, left to right: growth at −1 MPa, −2.5 MPa, and −5 MPa.

All Pds isolates were capable of germination and subsequent growth on autoclaved *Locusta migratoria* (insect), *Poecilia* sp. (fish), and *Lentinula edodes* (mushroom) within 30 days (Figure 1A–C). Germination and subsequent growth was visually observed on demineralized *Pleoticus muelleri* Bate (shrimp) exoskeletons, while minimal growth was observed on the demineralized and deproteinated exoskeletons under 10× microscopic examination (Figure 1D). Gelatin liquefaction and alkalinization occurred on both 3% and 6% gelatin, and no decrease in pH was observed prior to alkalinization on the 6% gelatin medium. All Pds isolates grew well on α-keratin azure medium, but no keratinolytic activity (dye release) was visually present (Figure 1E) and no growth was evident on the β-keratin mat (Figure 1F). The addition of sodium thiosulfate and sodium sulfite to the keratin azure medium did not result in the ability of Pds to degrade keratin at pH 7.5. Beta-glucosidase activity was positive within 1–2 days, while Mn-dependent peroxidase and chitinase activity were negative (Figure 1G). Lipase activity was positive on both olive oil and lard, and lipase/esterase activity was observed through the presence of calcium fatty acid crystals (Figure 1H).

### Nitrogen

Nitrogen assays demonstrated growth on multiple nitrogen sources (nitrate, nitrite, ammonium, and L-asparagine) indicating a Class 2 nitrogen profile for all Pds isolates. However, mycelial growth was influenced by both pH and sole nitrogen source (Figure 1K). Growth on nitrate was better at lower pH values (5–7), while growth on L-asparagine and ammonium was greatest at higher pH values (7–8). Growth occurred on nitrite by day 20, but only at pH 7 and pH 8. Growth was minimal on uric acid. The urease assay was only positive on media that contained urea, with positive results typically within 1–2 days on Christianson's media and within 3–4 days on Stewart's buffered medium.

### Calcium, pH, and sulfur tolerance

**Figure 2:**
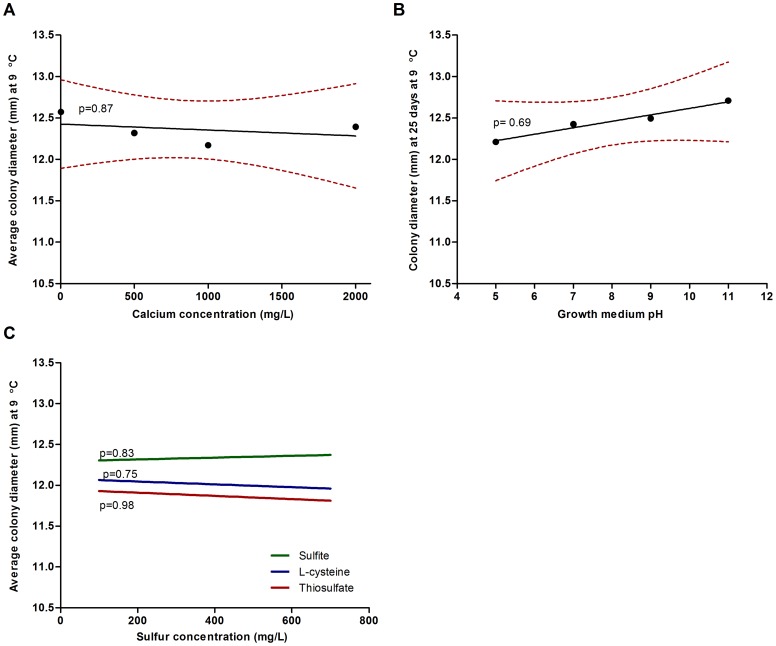
*Pseudogymnoascus destructans* tolerance to calcium, pH, and three sulfur compounds. A) one-way ANOVA analysis indicated no significant difference (p = 0.87) in radial colony diameter on PDA amended up to 2000 mg/L of calcium chloride. B) one-way ANOVA analysis indicated no significant difference (p = 0.69) in the radial colony diameter over the pH range of 4–11. C) one-way ANOVA analysis indicated no significant difference in radial colony diameter on PDA amended with 200–700 mg/L of sodium sulfite (p = 0.83), L-cysteine (p = 0.75), or sodium thiosulfate (p = 0.98). Panel A and B dotted line refers to 95% confidence interval, panels A–C solid line refers to best fit line of mean values.

All isolates were identical in their tolerance to pH (Figure 1I), calcium (Figure 1J), sodium thiosulfate, L-cysteine, and sodium sulfite. Using ANOVA analysis with Tukey's post test, the combined results of all six Pds isolates demonstrated no significant difference (p = 0.87) in colony radial diameter over the calcium range of control (20 mg) to 2000 mg/L and no significant difference (p = 0.69) in colony radial diameter was evident over a wide pH (5–11) range (Figure 2A–B). *Pseudogymnoascus destructans* isolates demonstrated tolerance to all three sulfur compounds with no significant difference in the combined colony radial diameter over the range of 100 mg/L to 700 mg/L for sodium thiosulfate (p = 0.98), L-cysteine (p = 0.75), and sulfite (p = 0.83, Figure 2C). In addition, Pds was equally tolerant (p = 0.13) to all three sulfur compounds. Hydrogen sulfide production was evident on PDA amended with sodium thiosulfate, while no hydrogen sulfide was evident on L-cysteine or sodium sulfite amended PDA.

### Matric potential and surface tension

Visible growth occurred initially where water was under minimal matric force (−0.07 MPa), while greatest biomass by day 25 occurred in the −1 MPa medium. Delayed germination and subsequent growth occurred at −2.5 MPa by day 25, with no visible growth occurring at −5 MPa. In contrast to the matric potential assay, growth occurred in all surface tension experiments (−0.07 to −5 MPa) (Figure 1L–M). ANOVA-analysis with Tukey's post test indicated no statistical difference in surface tension (p = 0.6) between the −1 MPa control medium and −1 MPa media exposed to two different Pds isolates.

## Discussion

### Keratinaceous substrates

Keratinaceous substrates containing carbon found in caves include bird feathers and mammal hair and skin. Good indicators in favor of Pds having some keratinolytic activity include the ability to grow on a sole protein source [19], tolerant of high pH and high environmental levels of L-cysteine and sulfite [37], ability to degrade gelatin in conjunction with environmental alkalinization [21], and the ability to convert thiosulfate to hydrogen sulfide (a reducing agent of keratin) [38]. However, no *in vitro* demonstration of keratinolytic activity was seen on keratin azure (with and without an environmental reducing agent) or the soluble β-keratin derived substrate (Figure 1E–F). Unfortunately these results do not resolve whether Pds is keratinophilic or actually keratinolytic. Keratinolytic ability may be difficult to demonstrate *in vitro* because keratinaceous materials contain significant amounts of non-keratin proteins and lipids [39], [40] and fungi tend to use more readily accessible non-keratin carbon sources prior to reverting to more energetically intensive resources [21]. Our results suggest that keratinaceous substrates (such as bat skin) may show signs of structural degradation without explicit keratinolytic activity. Regardless of whether Pds is keratinophilic or keratinolytic, it is capable of generating a micro-environment in which keratinaceous substrates found in cave soils are more susceptible to degradation and can serve as an important resource for Pds.

### Chitinaceous substrates

Arthropod (insects, crustaceans, arachnids, and myriapods) remains, undigested insect parts in guano, and fungal cell wall fragments offer another complex and variable carbon-containing substrate found in cave environments. Major components found in arthropod exoskeletons are proteins and chitin, with small amounts of lipids [41], [42], while fungal cell walls contain greater than 90% polysaccharides consisting of glucans, mannans, and chitin [43] and fungal basidiocarps can contain between 7.8 to 21% lipids [44]. To elucidate which structural components Pds isolates could utilize in chitin-containing substrates, we preformed three separate assays using shrimp exoskeletons, autoclaved fungal basidiocarps, and colloidal chitin. The shrimp exoskeletons were demineralized to obtain a substrate that would be similar to bat digested insect exoskeletal remains found in guano. The demineralization process was consistent with food passage times for several bat species including *Myotis lucifugus* [45]. Additional shrimp exoskeletons received a subsequent deproteination step to obtain a high chitin/low protein substrate. All Pds isolates grew well on demineralized shrimp exoskeletons, but microscopic examination was needed to visualize growth on deproteinated shrimp exoskeleton remains (Figure 1D). Similarly, Pds could grow on autoclaved *L. edodes* basidiocarps (Figure 1C), but on colloidal chitin Pds did not demonstrate a clearing zone (chitinase activity) and growth was sparse (Figure 1G). These results strongly suggest that Pds cannot degrade chitin, but rather Pds utilizes other nutritional components found in chitinaceous substrates. Similar to keratinaceous substrates, chitinaceous substrates are important resources for Pds due to the presences of other nutritional component (proteins and lipids).

### Cellulosic substrates

The genus *Pseudogymnoascus* is currently included in the fungal family *Pseudoeurotiaceae* Malloch & Cain of which many members grow saprotrophically on woody tissue [20]. Research has shown that many confounding variables (temperature, substrate structure and compositions, and physiological conditions) exist when assaying fungi for cellulolytic activity [37]. Because wood degradation has many confounding variables, we focused our efforts on detecting β-glucosidase and Mn-dependent peroxidase activity. All Pds isolates demonstrated a strong positive reaction for β-glucosidase activity within 24–48 hours of transfer to esculin iron media suggesting that β-glucosidase is constitutive (Figure 1G). Manganese-dependent peroxidase was negative and no noticeable increase in hyphal or medium pigmentation occurred (Figure 1G). Since most members within the family *Pseudoeurotiaceae* grow saprotrophically on woody tissues and rotting vegetation and Pds produces β-glucosidase, it is highly probable that Pds can degrade cellulosic substrates. Recent research supports this conclusion by demonstrating that Pds could penetrate dead moss cell walls [14]. We conclude that cellulosic substrates could be potential substrates for Pds, but cellulosic materials may not prove to be adequate for long-term substrate colonization. Based on our matric-induced water stress results and the fungal cell wall attributes of psychrophilic fungi [46], in order for cellulosic substrate to be utilized by Pds, the substrate would need to have sufficient moisture to reduce the water potential of cellulosic debris [47]. Consequently, cellulosic substrates would be unsuitable for long term colonization by Pds in caves or portions of caves that have frequent moisture fluctuations.

### Protein/lipid rich substrates

Many complex substrates (bat skin, arthropod exoskeletal and fish remains) contain proteins, lipids and esters as previously discussed. Fish are generally composed of 15–30% protein, 0–25% fat, and 50–80% water [48], while fish scale composition can vary. All Pds isolates produced ample mycelial growth and conidiation on the whole fish substrate (Figure 1A), as well as whole insect (Figure 1B) and fungal basidiocarps (Figure 1C). Pds isolates could degrade olive oil, lard, and Tween 80 media with positive reactions occurring within one week (Figure 1H). Our positive lipase and esterase results are consistent with previously published results [11]. In addition, Pds clearly was capable of utilizing gelatin as a sole source of nutrition. Taken together, the lipid and protein results indicate that Pds is capable of utilizing many lipid and protein sources found in complex carbon substrates. Unfortunately for hibernating bats, their skin and glandular secretions contain many proteins and lipids [49] making them good substrates for Pds.

### Nitrogen utilization

Guano, ammonium, urea, and bacterial fixation account for some of the nitrogen resources available within caves, but most cave nitrogen enters in a dissolved state in run-off water or leeching ground water [50]. *Pseudogymnoascus destructans* isolates demonstrated a rapid positive urease result usually within 24–48 hours, which suggests that urease is constitutive. *Pseudogymnoascus destructans* can utilize many different sources of nitrogen (Figure 1K); however the uric acid assay was weakly positive indicating that it may be a poor nitrogen resource due to low solubility [51]. Overall, our results indicate that in neutral to alkaline environments, nitrate, nitrite, ammonium, and amino acids sources are all sufficient for good growth, while uric acid is a potential nitrogen resource under alkaline conditions. Our results are in agreement with similar nitrogen studies using basidiomycetes [30], *Penicillium*, *Aspergillus* [52] and *Monascus* species [53] and reflect the importance of how pH interacts with nitrogen uptake. Importantly, Pds has demonstrated urease activity which has been proposed as a dual use virulence factor in the pathogenesis of *Cryptococcus neoformans* (San Felice) Vuill. and other pathogenic fungi [12], [54].

### Calcium, pH, and sulfur tolerance

Environmental chemistry can influence fungal growth and its ability to successfully colonize substrates [55]. Carbon and nutrient availability and the solubility of metals are influenced by soil pH [56] with high levels of environmental sulfur compounds capable of inhibiting fungal growth [57], while calcium has been shown *in vitro* to enhance the growth and reproduction of many fungi [18]. The growth and sporulation of Pds was not affected by the addition of calcium (2000 mg/L), which suggests that environmental calcium levels do not significantly impact substrate suitability (Figure 2A). Similarly, substrate pH assays showed that Pds is alkali-tolerant and capable of uniform growth from pH 5 to pH 11 (Figure 2B), which is also partially supported by the nitrogen assays (ph 5–8). The growth and sporulation of Pds isolates were not inhibited by 700 mg/L of sodium thiosulfate, sodium sulfite, or L-cysteine (Figure 2C) and hydrogen sulfide production was only detected in thiosulfate amended medium. The results of the pH and sulfur compound assays suggest that Pds has the potential to inhabit most caves and cave substrates since neither pH nor inhibitory sulfur compounds found in soils, urine, and bat skin appear to limit substrate suitability.

### Water interactions

The properties of water at low temperatures (i.e. increased viscosity and surface tension) and water availability are important abiotic factors that influence substrate/fungal interactions and can represent major challenges for habitat colonization [35], [58]. To investigate Pds tolerance to substrate matric potential, we utilized PEG 8000 since it is non-toxic and not metabolized by fungi [59] and is analogous to soil colloids [60]. Matric potential is the ability of the substrate to hold onto water through the combination of capillary and particle adsorptive forces [61] and is predominant in unsaturated soils [62]. Cave soil samples from four Illinois Pds positive caves were analyzed for matric potential and hydroscopic moisture content in order to relate our *in vitro* water interaction experiments to known Pds positive caves. The average Illinois Pds positive cave soil hydroscopic moisture content was 12.41±7.572% indicating an unsaturated environment with a matric potential range of −0.005 to −1.26 MPa, although spatial and temporal heterogeneity is to be expected. When exposed to PEG-induced water stress (matric potential), all Pds isolates conformed to the Type II response [63] (greater growth under minimal matric stress with a reduction of growth as matric stress increases), which is typical of most soil fungi [46] (Figure 1L). The matric potential results are supported by the Illinois cave soil matric potential analysis since Pds spore germination and active growth occurred within a similar range. In addition, our matric potential results are supported by the cell wall physiology of psychrophilic fungi; psychrophilic fungi have a greater degree of unsaturated lipids in their cell wall which is reported to reduce the ability of fungi to tolerate water stress [46]. Interestingly, Pds isolates were capable of growth and sporulation up to and including −5 MPa medium by the addition of 0.0125% Tween 80 (Figure 1M). This result is consistent with the fact that a reduction in surface tension reduces the capillary forces [62] and, consequently, reduces the overall matric force of the medium. The combination of these two results suggests that Pds is sensitive to matric-induced water stress. The surface tension comparison of media with and without exposure to Pds indicated that Pds was not capable of producing any extracellular biosurfactants or surface tension reducing compounds. Collectively, these three assays suggest that substrates containing surface tension reducing agents would be beneficial for the growth of Pds. In the cave environment, surface tension reducing substrates would contain free fatty acids or lipids that could be hydrolyzed to fatty acids by Pds. The skin of bats contains associated lipids and its glandular secretions contain free fatty acids [49] making the surface of bat skin excellent habitats for Pds.

## Conclusions

The examination of six isolates from four Eastern and Midwestern states demonstrated that Pds possesses Class 2 nitrogen utilization, a Type II response to matric stress, is alkali-tolerant, and is capable of saprobically utilizing many complex carbon-containing cave substrates. Based on current biological information, temperature and water availability are the only environmental limitations of a cave, mine, or similar environment to act as a reservoir for Pds. Our study of biotic and abiotic factors suggests that Pds would be able to remain in previously infected caves as a saprobe using small complex organic carbon-containing substrates that act as resource islands by limiting biotic competition. Due to the slow growth characteristics of Pds, it is likely that rapid and copious reproduction via conidiation occurs as a way to island hop, or bridge the gap between suitable resource islands. Although cave biotic and abiotic factors may limit accessibility or assist/hinder substrate degradation that cannot be reproduced *in vitro*, more research is needed on the basic biology and ecology of Pds since it will likely become a permanent resident in the majority of North American cave ecosystems.
